# Impact of chest computed tomography-determined low skeletal muscle mass on the survival of patients with acute heart failure

**DOI:** 10.3389/fcvm.2025.1569681

**Published:** 2025-08-04

**Authors:** Yan Huang, Yiming Wang, Junfang Guo, Ping Fang, Biao Kong, Zilan Wang, Junbo Zuo

**Affiliations:** ^1^ Department of Cardiology, The Affiliated People’s Hospital of Jiangsu University, Zhenjiang, China; ^2^ Department of Clinical Nutrition, The Affiliated People’s Hospital of Jiangsu University, Zhenjiang, China; ^3^ Department of General Surgery, The Affiliated People’s Hospital of Jiangsu University, Zhenjiang, Jiangsu, China

**Keywords:** computed tomography, acute heart failure, skeletal muscle mass, skeletal muscle index, prognosis

## Abstract

**Background:**

Computed tomography (CT)-determined low skeletal muscle mass (SMM) has been reported to be associated with poor clinical outcomes in various diseases; however, limited information is available regarding patients with heart failure (HF). Our research aimed to investigate the prognostic significance of low SMM assessed by chest CT scans in hospitalized patients with acute heart failure (AHF).

**Methods:**

Skeletal muscle index (SMI) was assessed using chest CT scans at the twelfth thoracic vertebra (T12) level, and sex-specific optimal cutoff values for T12 SMI were determined using the X-tile program based on all-cause mortality. The outcomes of this study were all-cause death and cardiovascular death. Cox proportional hazards models were employed to identify the risk factors for mortality.

**Results:**

This study enrolled 305 inpatients with AHF (62.3% males). According to the optimum cutoff value (31.9 cm^2^/m^2^ for females and 40.6 cm^2^/m^2^ for males), a total of 154 patients (50.5%) had low SMM. Kaplan–Meier survival analysis showed that patients with low SMM had a higher likelihood of experiencing all-cause death (*p* < 0.001) and cardiovascular death (*p* < 0.001) compared to those with normal SMM. Furthermore, multivariable Cox regression analysis revealed that low SMM was independent risk factors associated with all-cause death [hazard ratio (HR) = 2.37, 95% CI: 1.51–3.73; *p* < 0.001] and cardiovascular death (HR = 3.06, 95% CI: 1.75–5.37; *p* < 0.001).

**Conclusions:**

Chest CT-determined low SMM has the potential to serve as a valuable imaging prognostic indicator for predicting adverse outcomes in AHF patients.

## 1 Introduction

Heart failure (HF) is a widespread global epidemic that represents an advanced stage or severe manifestation of various heart conditions. Its prevalence significantly increases with age progression. Despite substantial advancements in both pharmaceutical and non-pharmaceutical therapies, the prognosis remains unfavorable. It continues to be a primary cause of mortality and morbidity worldwide, imposing significant financial burdens on patients, families, and healthcare systems (1).

Sarcopenia is a progressive systemic skeletal muscle disease, characterized by the accelerated loss of muscle mass, strength, and function (2), which is often worsened by HF (3). This can lead to decreased physical performance, lower quality of life, and an increased risk of disability, falls, and mortality (2, 4). Therefore, it has been identified as a strong predictor of adverse outcomes in various clinical scenarios (5–7). The occurrence of sarcopenia in patients with HF has been reported to range from 10% to 69% in previous studies (8). Studies have indicated that sarcopenia is the primary indicator of poor clinical outcomes in patients with HF, including a poor quality of life, longer hospital stays, recurrent hospitalizations, as well as an increased risk for mortality (9, 10). Consequently, it is recommended that the assessment of sarcopenia should be integrated into standard care protocols for patients with HF in clinical settings (11). However, there is currently no standardized method tailored to HF for identifying muscle wasting, and there are no established normal reference values for this particular population (12). Therefore, it is crucial to develop a reliable and convenient approach for assessing the quality and quantity of skeletal muscles mass (SMM), allowing for early identification of sarcopenia in patients with HF.

CT imaging technology is widely regarded as the promising method for precise measurement of total body and regional SMM (13). Research has widely utilized single-slice CT scans at the third lumbar vertebra (L3) level to measurement SMM, which have shown correlations with adverse clinical outcomes in various diseases (14, 15). However, chest CT scans are the preferred tool in HF due to their more frequent utilization in routine clinical practice compared to abdominopelvic CT scans. Currently, single-slice CT scans at the twelfth thoracic vertebra (T12) level have been validated as a valuable tool for evaluating SMM. Furthermore, recent research has demonstrated a robust association between skeletal muscle index (SMI) at the T12 level (T12-SMI) and L3-SMI in clinical settings, with comparable prognostic relevance in clinical contexts (16–18).

Nevertheless, there is limited available information regarding the association between SMM determined by chest CT and clinical outcomes in patients with HF. Therefore, the objective of this study was to investigate the association between chest CT-determined low SMM and prognosis in AHF patients.

## 2 Materials and methods

### 2.1 Study population

The present study was a retrospective cross-sectional analysis of consecutive patients with AHF who were admitted to the Department of Cardiology, People's Hospital Affiliated to Jiangsu University between May 2020 and December 2021. AHF encompasses the onset of new HF as well as the acute worsening of pre-existing chronic HF. The inclusion criteria were as follows: (1) age ≥18 years; (2) diagnosis of AHF based on the 2021 ESC guidelines for the diagnosis and treatment of acute and chronic HF (19). The patients were further excluded based on the following criteria: (1) presence of cancer; (2) presence of liver cirrhosis; (3) undergoing hemodialysis treatment; (4) in-hospital death; (5) absence of chest CT images or poor-quality CT images; (6) loss of follow-up. This study adhered to the Declaration of Helsinki and received approval from the Ethics Committee of The Affiliated People's Hospital of Jiangsu University (No. K-20240094-W). The requirement for written informed consent was eliminated due to the retrospective design.

### 2.2 Measurement of skeletal muscle mass and quality

The non-enhanced chest CT examinations were conducted within 48 h of hospital admission using the 64-layer spiral CT (SOMATON sensation64, SIEMENS Healthcare, Germany) or 256-layer spiral CT scanners (Brilliance iCT, ROYAL PHILIPS, Netherlands). A senior radiologist extracted axial CT slices of the T12 vertebra level for each patient from the medical imaging management system, followed by independent analysis of the images by two trained observers using the image analysis software Slice-O-matic (Tomovision, version 5.0, Magog, Quebec, Canada). They were blinded to the patients' clinical outcomes when reviewing all selected CT slices and determining the SMM area. The pre-validated CT threshold of −29 to +150 Hounsfield units (HU) was utilized for identification and quantification all skeletal muscles in the selected axial images (20). The cross-sectional area of skeletal muscle tissue (cm^2^) at T12 was automatically calculated by the software and standardized as T12 SMI (cm^2^/m^2^) by dividing skeletal muscle area by height squared (Supplementary Figure S1) (21). The skeletal muscle density (SMD) in HU was determined by calculating the average radiodensity of the entire muscle area at T12 cross-sectional levels.

The inter-observer variability was assessed in 30 randomly selected scan samples. The intraclass correlation coefficient values were employed to demonstrate the consistency among observers when evaluating the skeletal muscle area.

Interobserver variability was evaluated in 30 randomly selected samples from the scans. The intraclass correlation coefficient (ICC) was used to evaluate the consistency among observers when measuring the area of skeletal muscle. As the measurements were semi-automated, no assessment of intra-observer variability was performed. As shown in Supplementary Figure S2, the optimal cutoff value of T12 SMI for both male and female patients was determined using the X-tile program based on all-cause death (22). According to the sex-specific optimal cutoff value, patients were stratified into two groups: low SMM group and normal SMM group.

### 2.3 Data collection

We obtained patients' personal data from the electronic medical record system, including demographic information (sex and age), anthropometric measurements (height and weight), lifestyle factors (smoking and drinking history), medical history (hypertension, diabetes, coronary heart disease), clinical laboratory test results [hemoglobin, liver and kidney function tests, uric acid, fasting blood glucose and lipid, albumin and B-type natriuretic peptide (BNP) ], 12-lead electrocardiogram findings, thoracic echocardiography results, as well as medications used at discharge. Clinical laboratory results within 24 h of admission were collected. The BNP analysis was performed using a commercially available immunoassay (Alere Inc., Waltham, MA) on the Alere Triage MeterPro analyzer, following the manufacturer's instructions. BNP values were transformed into a logarithmic scale (log BNP) for statistical analysis. According to the left ventricular ejection fraction (LVEF), we further classified the patients into heart failure patients with reduced ejection fraction (HFrEF, LVEF <50%) and those with preserved ejection fraction (HFpEF, LVEF ≥50%). The estimated glomerular filtration rate (eGFR) [ml/min/1.73m^2^] can be calculated using the Cockcroft-Gault formula: [(140−age)×weight(kg)]÷[0.818×Scr(umol/L)] for males and [(140−age)×weight(kg)×0.85]÷[0.818×Scr(umol/L)] for females (23). The definition of anemia involves a hemoglobin level below 12 g/dl for males and below 11 g/dl for females. Hypoalbuminemia is characterized by a serum albumin level lower than 3.5 g/dl. Hyperuricemia is identified as a serum uric acid concentration exceeding 7.0 mg/dl.

### 2.4 Survival follow-up and endpoint events

All patients received standard heart failure treatment after discharge. The endpoint events of this study were all-cause mortality and cardiovascular mortality. Cardiovascular death primarily refers to mortality caused by congestive HF, malignant arrhythmia, myocardial infarction, sudden death, stroke, or another cardiac-related issue. The late follow-up was conducted in April 2024. Survival data were collected from the electronic medical record system by a clinician or through telephone contact with patients or their family members.

### 2.5 Statistical analysis

All statistical analyses were performed using IBM SPSS Statistics for Windows, version 25.0 (IBM Corp., Armonk, NY, USA). The distribution of continuous variables was examined using the Kolmogorov–Smirnov test. Continuous data were presented as mean ± standard deviation (SD) or median and interquartile range (IQR) for normally and not normally distributed continuous variables, respectively. Group differences were compared using appropriate statistical tests such as Student's *t*-test or Mann–Whitney *U* tests. Categorical variables were described as numbers and frequencies (%), with Chi-squared tests applied for analysis. Spearman's rank correlation analysis (*r*) was used to investigate the association between SMI and clinical indicators. The Kaplan–Meier analysis with log-rank test was utilized to evaluate survival, while univariable and multivariable Cox regression analyses were further employed to identify risk factors associated with mortality. The presence of multicollinearity among variables was assessed using the Variance Inflation Factor (VIF), where variables with a VIF value ≥10 were subsequently excluded from further analysis. Hazard ratio (HR) was calculated and the results were presented as HR with a 95% confidence interval (CI). The subgroup analyses were conducted based on sex, age (<65 years or ≥65 years), BMI (<24 kg/m^2^ or ≥24 kg/m^2^), NYHA classification, hypertension, diabetes, ischemic etiology, HF phenotypes (HFrEF or HFpEF) and atrial fibrillation. The likelihood ratio test was employed to evaluate the interaction in these subgroups. Statistical significance was determined at a two-tailed *p* value <0.05 for all analyses conducted in this study.

## 3 Results

### 3.1 Patient characteristics

**Figure 1:**
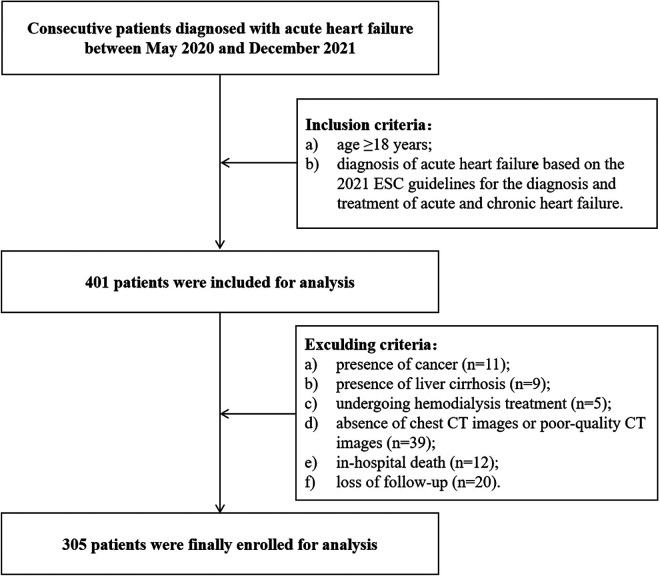
Flow diagram of the patient inclusion and exclusion process of this study. CT, computed tomography.

After implementing rigorous exclusion criteria, a total of 305 patients with AHF were included in this study (Figure 1). The demographic and clinical characteristics of the participants are shown in Table 1. Among them, there were 190 (62.3%) males and 115 (37.7%) females. The median age was 70 years (IQR: 61–77), and the median BMI was 23.66 kg/m^2^ (IQR: 21.97–25.89). The sex-specific cut-off values of T12 SMI associated with all-cause death, as determined by the X-tile program, were 40.6 cm^2^/m^2^ for males and 31.9 cm^2^/m^2^ for females, respectively. Based on these defined thresholds, 154 (50.5%) patients had low SMM. Patients with low SMM were older [74 (IQR: 68–79) vs. 66 (IQR: 54–73), *p* < 0.001], more likely to be male (70.1% vs. 54.3%, *p* = 0.004), had a greater proportion of ischemic etiology (52.0% vs. 33.8%, *p* = 0.001) and NYHA class III–IV (85.7% vs. 62.3%, *p* < 0.001), and a longer median LOS [8 (IQR: 7–11) days vs. 7 (IQR: 5–8) days, *p* < 0.001] compared to those with normal SMM. Additionally, these patients showed lower levels of body mass index (BMI), hemoglobin, eGFR, albumin, cholesterol and triglyceride levels, but higher levels of log BNP and uric acid (all *p* < 0.05). The phenotypic disparities classified according to LVEF indicated that 230 (75.4%) patients had HFrEF (LVEF < 50%), while 75 (24.6%) patients had HFpEF (LVEF ≥ 50%). No association was found between HF phenotype and low SMM.

**Table 1:** Baseline characteristics of the study population.

Characteristic	Total (*n* = 305)	Low SMM (*n* = 154)	Normal SMM (*n* = 151)	*P*-value
Age, year	70 (61–77)	74 (68–79)	66 (54–73)	<0.001
Sex, *n* (%)				0.004
Male, *n* (%)	190 (62.3)	108 (70.1)	82 (54.3)	
Female, *n* (%)	115 (37.7)	46 (29.9)	69 (45.7)	
BMI, kg/m^2^	23.7 (22.0–25.9)	22.7 (20.9–24.2)	25.0 (23.0–27.3)	<0.001
SMD, HU	29.1 ± 7.1	27.9 ± 6.8	30.3 ± 7.2	0.003
Smoking, *n* (%)	136 (44.6)	72 (46.8)	64 (42.4)	0.443
Drinking, *n* (%)	59 (19.3)	25 (16.2)	34 (22.5)	0.165
Medical history, *n* (%)				
Hypertension	186 (61.0)	96 (62.3)	90 (59.6)	0.624
Diabetes	101 (33.1)	47 (30.5)	54 (35.8)	0.331
Atrial fibrillation	126 (41.3)	68 (44.2)	58 (38.4)	0.308
Etiology of ischemic, *n* (%)	131 (43.0)	80 (51.9)	51 (33.8)	0.001
Echocardiographic parameters				
LAD, mm	46 (43–50)	46 (42–50)	47 (43–51)	0.179
LVEF, %	37 (28–48)	37 (28–48)	35 (26.5–50)	0.605
LVEDD, mm	58 (51–65)	58 (50–63)	58 (52–65)	0.357
LVESD, mm	47 (37–55)	46 (37–54)	47 (37–56.5)	0.455
Heart failure phenotypes, *n* (%)				0.619
HFrEF (LVEF < 50%)	230 (75.4)	118 (76.6)	112 (74.2)	
HFpEF (LVEF ≥ 50%)	75 (24.6)	36 (23.4)	39 (25.8)	
NYHA functional class, *n* (%)				<0.001
II	79 (25.9)	22 (14.3)	57 (37.8)	
III	134 (43.9)	71 (46.1)	63 (41.7)	
IV	92 (30.2)	61 (39.6)	31 (20.5)	
Laboratory data				
BNP, ng/L	744 (346–1,300)	965 (489.5–1,582.5)	545 (284–1,008.5)	<0.001
Hemoglobin, g/dl	12.6 (11.3–13.8)	12.2 (10.8–13.6)	12.9 (11.9–14.4)	0.002
Anemia, *n* (%)	88 (28.9)	59 (38.3)	29 (19.2)	<0.001
CRP, mg/L	3.4 (1.1–8.7)	3.6 (1.5–11.4)	3.4 (0.7–7.2)	0.055
Albumin, g/dl	3.6 (3.3–3.9)	3.5 (3.2–3.8)	3.7 (3.4–4.0)	<0.001
Hypoalbuminemia, *n* (%)	121 (39.7)	75 (48.7)	46 (30.5)	0.001
eGFR, ml/min/1.73 m^2^	59.8 (43.7–79.5)	53.3 (38.1–73.2)	66.1 (48.4–91.8)	<0.001
Uric acid, mg/dl	7.4 (5.8–9.2)	7.9 (6.4–9.5)	6.9 (5.6–9.1)	0.010
Hyperuricemia, *n* (%)	163 (53.4)	92 (59.7)	71 (47.0)	0.026
Cholesterol, mmol/L	3.7 (3.1–4.6)	3.6 (3.1- 4.4)	3.9 (3.3–4.7)	0.014
Triglyceride, mmol/L	1.0 (0.8–1.4)	0.9 (0.7–1.2)	1.1 (0.8–1.5)	<0.001
LDL-C, mmol/L	2.0 (1.5–2.5)	1.9 (1.4–2.4)	2.0 (1.5–2.6)	0.147
Cardiovascular medications, *n* (%)				
ACE I/ARB/ARNI	222 (72.8)	107 (69.5)	115 (76.2)	0.190
Beta-blocker	224 (73.4)	108 (70.6)	116 (76.8)	0.186
Statin	201 (65.9)	106 (68.8)	95 (62.9)	0.276
Diuretics	290 (95.1)	150 (97.4)	140 (92.72)	0.058
MRA	263 (86.2)	135 (87.7)	128 (84.8)	0.463
LOS, day	7 (6–9)	8 (7–11)	7 (5–8)	<0.001

ACEI/ARB/ARNI, angiotensin-converting enzyme inhibitor/angiotensin receptor blocker/angiotensin receptor/neprilysin inhibitor; BMI, body mass index; BNP, B-type natriuretic peptide; CRP, C-reactive protein; eGFR, estimated glomerular filtration rate; HFpEF, heart failure with preserved ejection fraction; HFrEF, heart failure with reduced ejection fraction; HU, hounsfield units; LAD, left atrium diameter; LDL-C, low-density lipoprotein cholesterol; LOS, length of hospital stay; LVEDD, left ventricular end-diastolic diameter; LVESD, left ventricular end-systolic diameter; LVEF, left ventricular ejection fraction; MRA, mineralocorticoid receptor antagonist; NYHA, New York Heart Association; SMD, skeletal muscle density; SMM, skeletal muscle mass.

### 3.2 Consistency evaluation of skeletal muscle area measurement

The ICC for the inter-rater variability of skeletal muscle area measurements was 0.989 (95% CI: 0.965–0.998), demonstrating excellent consistency.

### 3.3 Correlation between T12 SMI and clinical parameters

**Figure 2:**
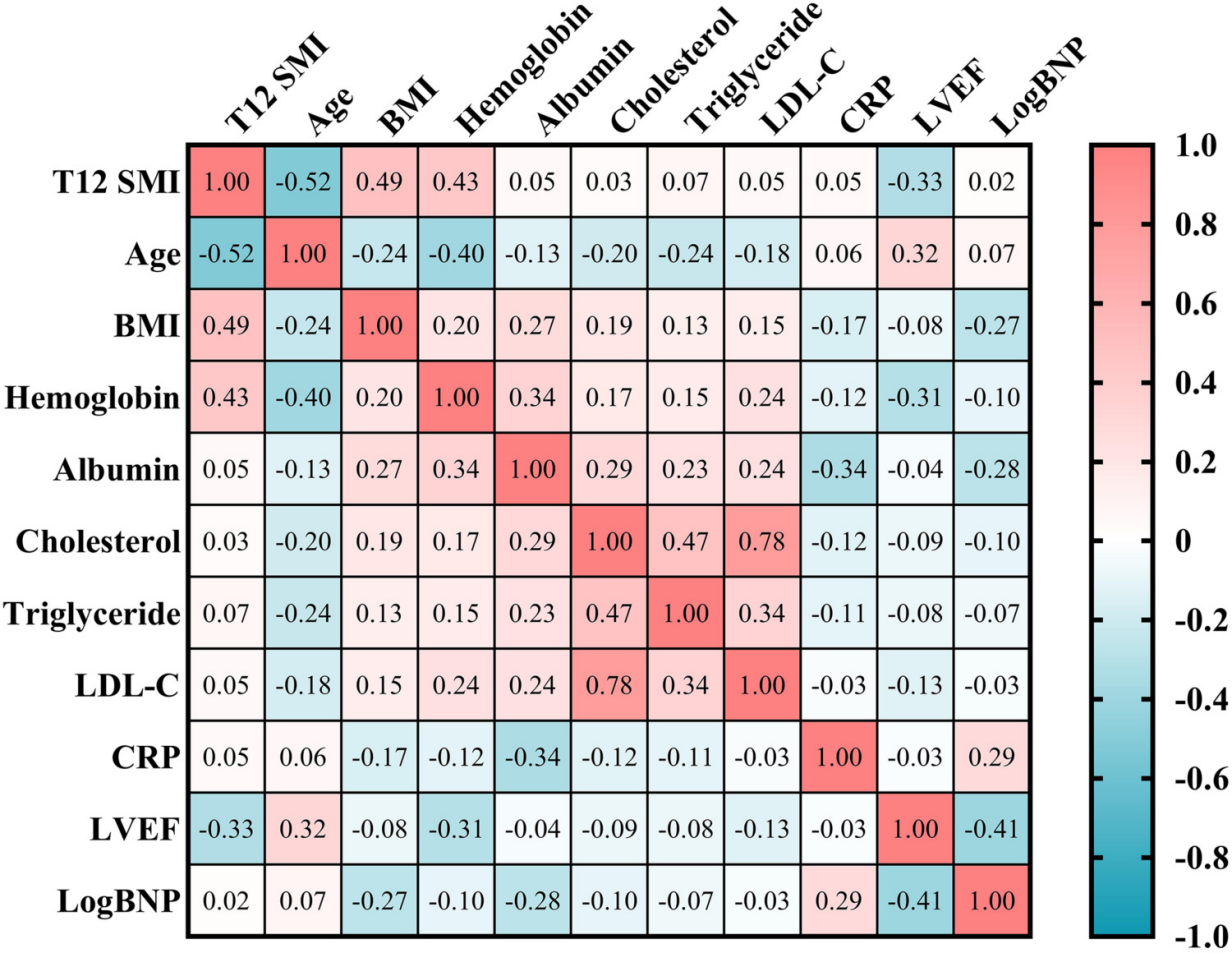
Spearman's correlation analysis of T12 SMI and clinical parameters. BMI, body mass index; BNP, brain natriuretic peptide; CRP, C-reactive protein; LDL-C, low-density lipoprotein cholesterol content; LVEF, left ventricular ejection fraction; T12 SMI, skeletal muscle index at the level of the twelfth thoracic vertebra.

Spearman's correlation analysis showed that T12 SMI was negatively correlated with age (*r* = −0.52, *p* < 0.001) and LVEF (*r* = −0.33, *P* < 0.001). Additionally, there is a strong positive correlation observed between T12 SMI and BMI (*r* = 0.49, *p* < 0.001) as well as hemoglobin (*r* = 0.43, *p* < 0.001). However, no significant correlations were found between T12 SMI and log BNP levels, albumin levels, cholesterol levels, triglyceride levels or CRP levels (Figure 2, all *p* > 0.05).

### 3.4 Risk factors for survival outcomes

**Figure 3:**
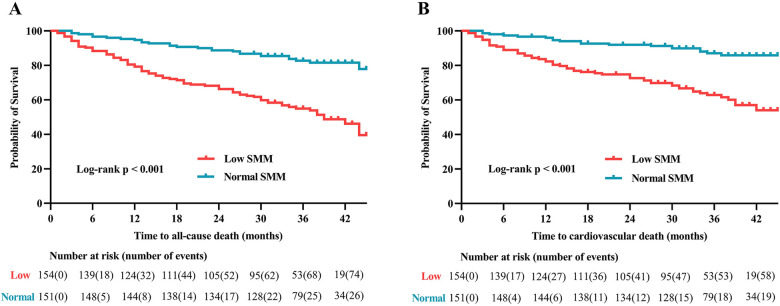
Kaplan–Meier analysis of **(A)** all-cause death and **(B)** cardiovascular death in patients with and without low SMM. SMM, skeletal muscle mass.

**Figure 4:**
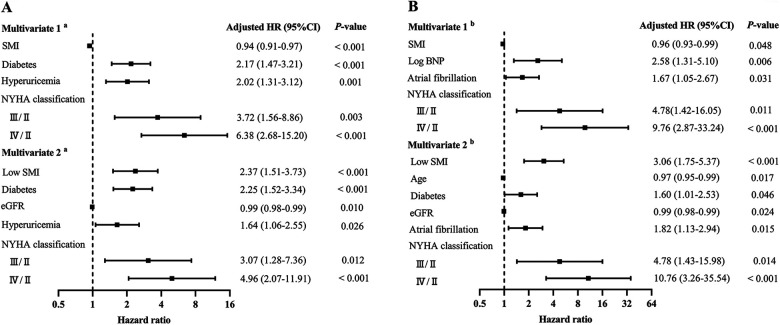
Forest plot of independent risk factors for all-cause death **(A)** and cardiovascular death **(B)**. BNP, B-type natriuretic peptide; eGFR, estimated glomerular filtration rate; NYHA, New York Heart Association; SMI, skeletal muscle index. “III/II” and “IV/II” represent hazard ratios for NYHA class III and IV compared to class II (reference group).

The median follow-up duration was 34 months (IQR: 27–39.5). Throughout the follow-up period, all-cause death occurred in 102 (33.4%) patients, while cardiovascular death was observed in 77 (25.3%) patients. According to Kaplan–Meier survival analysis, patients with low SMM had a higher likelihood of experiencing all-cause death (48.7% vs. 17.9%, log-rank *p* < 0.001; Figure 3A) and cardiovascular death (37.7% vs. 12.6%, log-rank *p* < 0.001; Figure 3B) compared to those with normal SMM. Based on the univariable Cox regression analysis presented in Supplementary Table S1, age, BMI, diabetes, atrial fibrillation, NYHA classification, log BNP, anemia, eGFR, hyperuricemia, and SMI (as both continuous and categorical variables) were identified as risk factors associated with all-cause death (*p* < 0.05). Meanwhile, BMI, atrial fibrillation, NYHA classification, log BNP, anemia, hypoproteinemia, eGFR, hyperuricemia, and SMI (as both continuous and categorical variables) were found to be risk factors for cardiovascular death (*p* < 0.05). In the multivariable Cox regression analysis, SMI as a continuous variable remained independently associated with all-cause death (HR = 0.94, 95% CI: 0.91–0.97, *p* < 0.001; Figure 4A, multivariate 1) and cardiovascular death (HR = 0.96, 95% CI: 0.93–0.99, *p* = 0.048; Figure 4B, multivariate 1). Furthermore, patients with low SMM exhibited a 2.37-fold increased risk of all-cause death (HR = 2.37, 95% CI: 1.51–3.73, *p* < 0.001; Figure 4A, multivariate 2) and a 3.06-fold increased risk of cardiovascular death (HR = 3.06, 95% CI: 1.75–5.37, *p* < 0.001; Figure 4B, multivariate 2) compared to those with normal SMM.

### 3.5 Subgroup analyses

**Figure 5:**
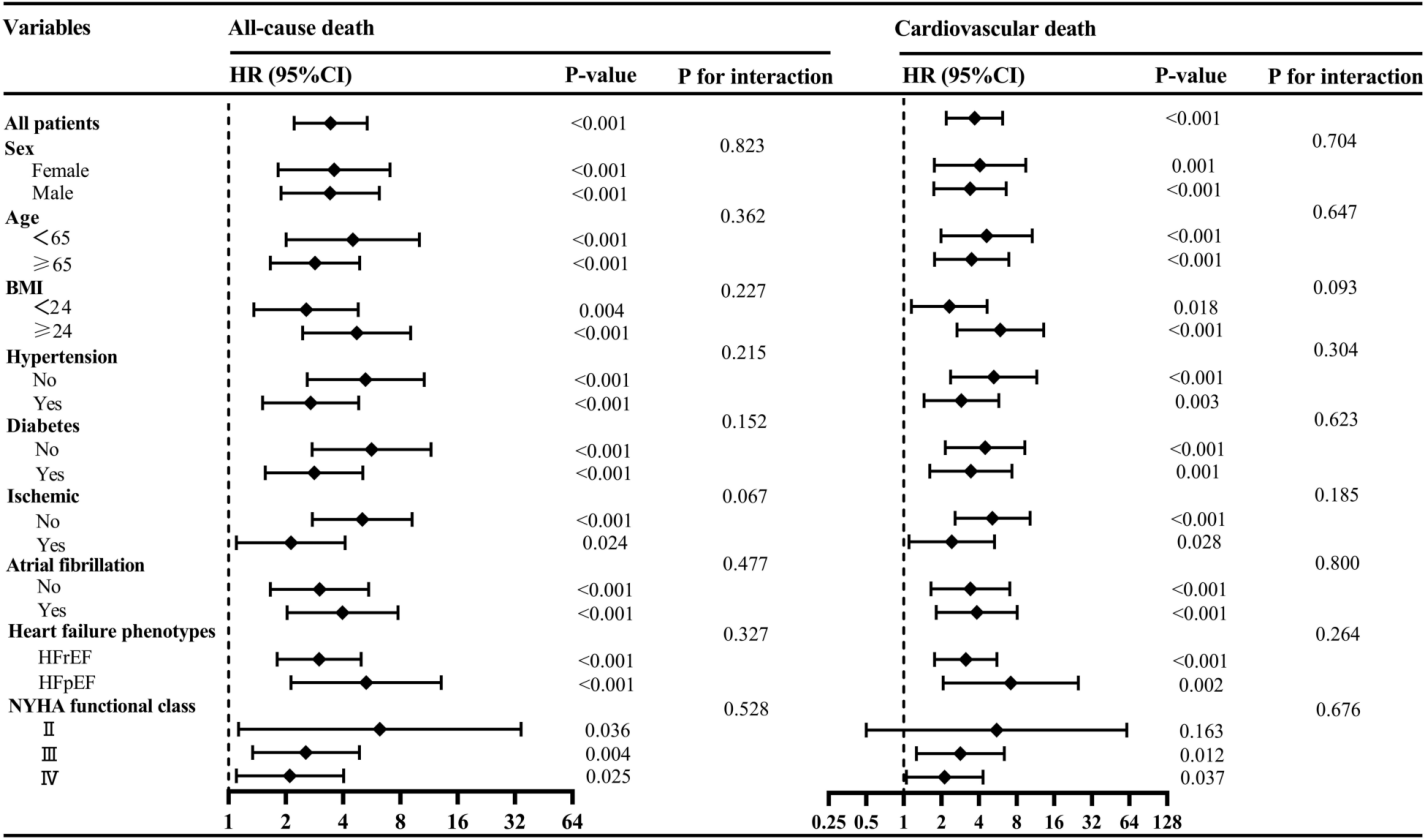
Subgroup analyses of the associations between low SMM and all-cause death and cardiovascular death. BMI, body mass index; HFpEF, heart failure with preserved ejection fraction; HFrEF, heart failure with reduced ejection fraction; NYHA, New York Heart Association; SMM, skeletal muscle mass.

To further investigate the association of low SMM with all-cause death and cardiovascular death across diverse population, we further conducted subgroup analyses based on sex, age, BMI, NYHA classification, hypertension, diabetes, ischemic etiology, HF phenotypes and atrial fibrillation. As depicted in Figure 5, no statistically significant interaction was observed (all *p* for interaction >0.05), indicating that the association between low SMM and all-cause death as well as cardiovascular death remained unaffected by these factors.

## 4 Discussion

Our study focused on utilizing clinically chest CT imaging to quantify SMM and investigate its prognostic implications in AHF patients. To the best of our knowledge, this is one of the few study that investigate the relationship between chest CT-determined SMM and survival outcomes in patient with AHF. In the present study, we found a significant and positive association between low SMM and increased all-cause mortality and cardiovascular mortality among AHF patients. Therefore, chest CT-determined low SMM may serve as a potentially reliable imaging biomarker for predicting adverse outcomes in AHF patients.

Muscle wasting is a significant concern not only for the elderly, but also a common complication of chronic diseases, independently associated with functional impairment, frailty, and mortality (2). A cross-sectional study utilizing the National Health and Nutrition Examination Survey database has demonstrated that low SMM is an independent risk factor for all-cause mortality and cardiovascular mortality in adults (24). Additionally, in certain specific populations such as those with chronic obstructive pulmonary disease, liver cirrhosis, and cancer, muscle wasting has been shown to be a stronger and independent predictor of adverse outcomes (6, 7, 25). Recent research has shown that muscle wasting is highly prevalent in patients with HF and has been recognized as a significant extracardiac factor that contributes to adverse clinical outcomes such as an increased likelihood of readmission for HF and higher mortality rates (12), despite variations in study populations and methodologies. In the studies investigating comorbidities aggravating heart failure, 200 stable outpatients with chronic HF and a disease duration of less than 3 months were included. SMM was assessed using dual-energy x-ray absorptiometry (DEXA), and muscle wasting was defined as a muscle mass index 2 SDs below the mean of a healthy young reference population aged 18–40 years. The results indicated that 19.5% of subjects had low SMM, which was associated with weaker muscle strength and reduced exercise capacity, as well as lower left ventricular ejection fraction (26). The prevalence of low SMM in our study cohort was significantly higher than that in this study, probably due to differences in the study population, as well as discrepancies in the measurement methods for SMM and the definition of low SMM. Another study conducted by Saito et al. investigated the prognostic significance of muscle mass in 226 elderly hospitalized patients with HF, using DEXA and bioelectrical impedance analysis (BIA). The study found poor consistency between the two methods of measuring SMM, and after adjusting for previous risk factors, only low SMM defined by DEXA, not BIA, was associated with all-cause mortality (27). This may be due to the susceptibility of BIA measurements to hydration status (28), thereby reducing the reliability of measurements in HF patients, which requires further investigation.

Currently, CT is a dependable technique favored by researchers for assessing muscle quantity and quality. Previous studies have fully confirmed that the muscle mass assessed by single-slice CT at L3 and T12 levels has predictive value for certain diseases (6, 15–18, 29). Chest CT has emerged as a valuable tool for diagnosing AHF due to its superior ability to detect subtle signs of congestion compared to chest x-rays and lung ultrasound (30). Its growing clinical utilization enables the acquisition of images at the T12 level in patients without additional expense. However, there remains a paucity of comprehensive research in the field of HF concerning the association between muscle mass evaluated through chest CT scans and clinical outcomes. Mirzai et al. conducted a retrospective study involving 385 patients with AHF and found that low SMM was independently associated with 1-year and 3-year all-cause mortality (31). Although the definition of low SMM in our study differs from theirs, the results are consistent with these observations, indicating that patients with low SMM have a higher risk of all-cause mortality and cardiovascular mortality. The potential reasons are as follows: low SMM may induce endothelial inflammation and exacerbate insulin resistance (32), thereby altering cardiac structure and function, ultimately leading to diastolic dysfunction (33). Moreover, low SMM is also associated with enhanced ergoreflex sensitization, which is considered an important contributor to typical symptoms of HF, such as exertional dyspnea, fatigue, and sympatho-vagal imbalance (34) Additionally, low SMM may also affect respiratory muscles, resulting in dyspnea, fatigue, and weakness, while worsening symptoms of exercise intolerance related to HF (35). This finding suggests that increasing muscle mass may be a feasible strategy to improve the prognosis of patients with AHF. Notably, exercise training, as an effective treatment for HF, can improve exercise tolerance by reducing ergoreflex sensitization and restoring the sympatho-vagal balance, further confirming the key role of ergoreflex sensitivity in the pathophysiology of HF in the context of low SMM (34). However, further comprehensive and rigorous intervention studies are required.

Serum albumin is a crucial biochemical marker for assessing nutritional status and metabolic function, with previous studies demonstrating their predictive value in HF (36). Armentaro et al. discovered that lower albumin levels in HF patients were linked to an increased risk of major adverse cardiac events, higher overall mortality, and a greater incidence of HF hospitalization (37). Similar findings were also reported by Ancion et al. (38). It is noteworthy that no correlation was observed between serum albumin levels and prognosis in this study cohort. This finding was incongruent with prior research results, possibly due to potential selection bias resulting from the inclusion criteria used in this study, thus limiting the generalizability of this finding.

This study has certain limitations: (1) Being a single-center cohort study, the relatively small sample size may introduce bias and confounding factors. Further validation through larger-scale multi-center studies is necessary to corroborate the results. (2) The X-tile program was employed to determine the optimal cut-off value based on all-cause death. While some subjectivity may exist in this process, there is currently no universally standardized cut-off value for CT-determined low SMM in patients with AHF. Nevertheless, we believe this method can provide valuable insights for future research. It is crucial to emphasize that the proposed SMM cut-offs are exploratory and require further validation in external cohorts. (3) While this study has indeed identified a new and promising alternative method for assessing sarcopenia, there is a lack of data on muscle strength and quality, such as grip strength and physical performance, which are essential for stratifying sarcopenia. Therefore, further research is necessary to address these gaps in the current understanding of the condition.

In conclusion, chest CT-determined low SMM at T12 level was independently associated with poor prognosis in patients with AHF. Utilizing chest CT for assessing muscle mass enables physicians to identify high-risk AHF patients and implement timely and effective intervention measures, guiding the selection of personalized treatment strategies to enhance patients' prognosis. Further research with larger prospective is urgently necessary to validate its efficacy and reliability.

## Data Availability

The original contributions presented in the study are included in the article/Supplementary Material, further inquiries can be directed to the corresponding author.
