# Supplementary material for: Impact of chest computed tomography-determined low skeletal muscle mass on the survival of patients with acute heart failure
Source: Front Cardiovasc Med. 2025 Aug 4;12:1569681. doi: 10.3389/fcvm.2025.1569681 (PMC12358454; doi:10.3389/fcvm.2025.1569681)
Supplement: Supplementary file 1 [file Datasheet1.pdf]

Table S1. Univariate Cox regression analysis for all-cause death and cardiac death

Covariables	All-cause death		Cardiovascular death	
	HR (95% CI)	*P*-value	HR (95% CI)	*P*-value
Age, year	1.03 (1.01-1.05)	<0.001	1.02 (1.00-1.04)	0.070
Sex, n (%)				
Male/ Female	1.07 (0.72-1.60)	0.737	1.27 (0.79-2.04)	0.328
BMI, kg/m^2	0.91 (0.86-0.97)	0.002	0.91 (0.85-0.97)	0.007
Smoking, n (%)	0.99 (0.67-1.46)	0.963	1.22 (0.78-1.91)	0.377
Drinking, n (%)	0.90 (0.54-1.49)	0.672	1.02 (0.58-1.79)	0.953
Hypertension, n (%)	0.75 (0.51-1.10)	0.144	0.70 (0.45-1.10)	0.120
Diabetes, n (%)	2.07 (1.40-3.06)	<0.001	1.57 (0.99-2.48)	0.053
Atrial fibrillation, n (%)	1.52 (1.03-2.24)	0.035	1.69 (0.08-2.65)	0.021
Ischemic etiology, n (%)	1.20 (0.81-1.77)	0.358	1.20 (0.81-1.77)	0.358
NYHA class, n (%)				
III/II	5.28 (2.25-12.37)	<0.001	6.95 (2.12-22.78)	0.001
IV/II	10.19 (4.37-23.77)	<0.001	17.39 (5.39-56.07)	<0.001
LVEF, %	1.00 (0.99-1.02)	0.601	0.99 (0.97-1.01)	0.246
Log BNP, ng/L	3.46 (2.05-5.85)	<0.001	5.18 (2.80-9.58)	<0.001
Hypoalbuminemia, n (%)	1.47 (1.00-2.18)	0.052	1.56 (1.00-2.44)	0.051
Anemia, n (%)	1.90 (1.28-2.82)	0.002	1.59 (1.01-2.54)	0.049
eGFR, mL/min/1.73 m^2	0.98(0.97-0.99)	<0.001	0.98 (0.97-0.99)	<0.001
Hyperuricemia, n (%)	2.40 (1.57-3.66)	<0.001	2.44 (1.50-3.99)	<0.001
SMD, HU	0.98 (0.96-1.01)	0.219	0.99 (0.96-1.02)	0.399
SMI, cm^2/m^2	0.93 (0.90-0.96)	<0.001	0.94 (0.90-0.97)	<0.001
Low SMI, n (%)	3.44 (2.21-5.35)	<0.001	3.69 (2.19-6.19)	<0.001

BMI, body mass index; BNP, B-type natriuretic peptide; eGFR, estimated glomerular filtration rate; HR, Hazard radio; HU, Hounsfield units; LVEF, left ventricular ejection fraction; NYHA, New York Heart Association; SMD, skeletal muscle density; SMI, skeletal muscle index.

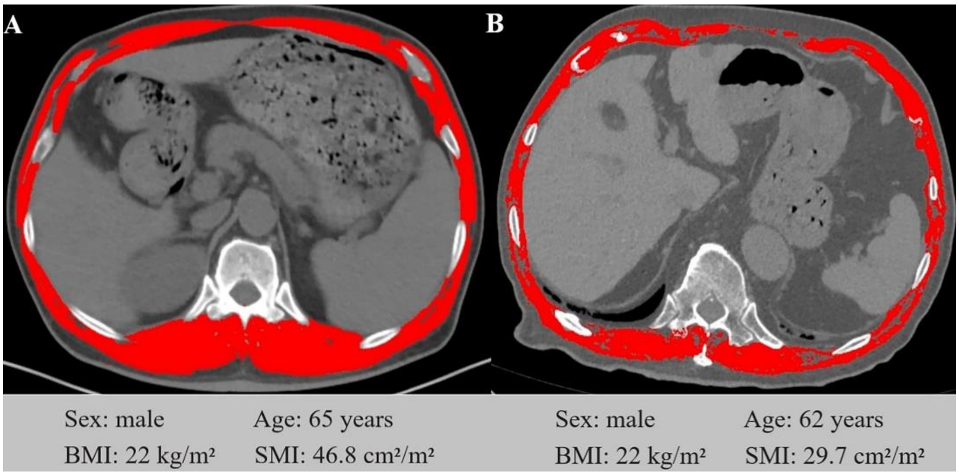

Figure S1. Computed tomographic scans of patients with AHF. (A) Patients with normal SMI; (B) Patients with low SMI; Red indicates skeletal muscle tissue at the level of the twelfth thoracic vertebra. AHF, acute heart failure; BMI, body mass index; SMI, skeletal muscle index.

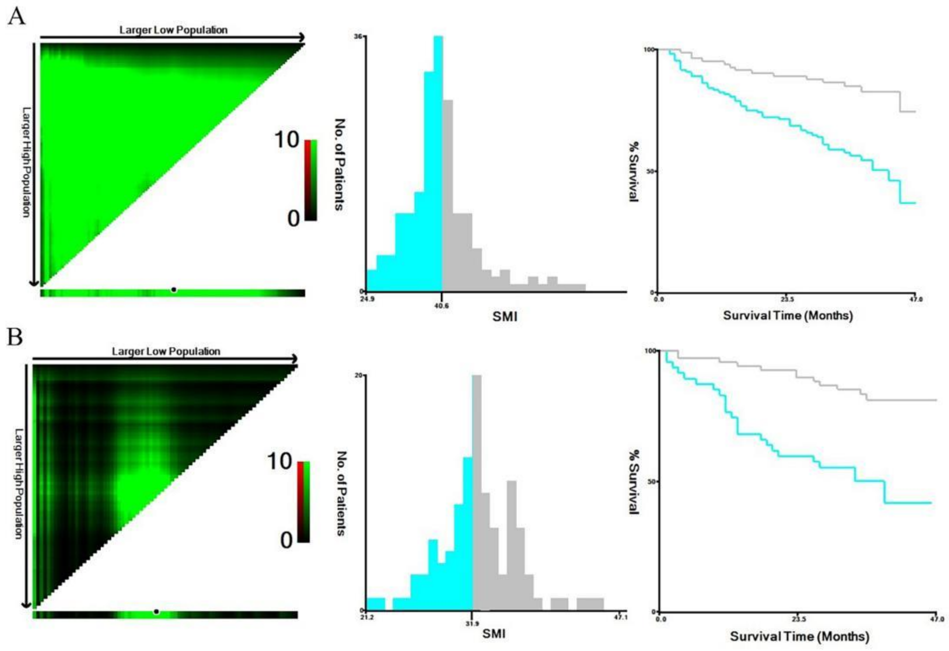

Figure S2. X-tile analyses of overall survival were conducted to determine sex-specific cut-off values of T12 SMI. (A) X-tile analyses for male; (B) X-tile analyses for female.
